# Supplementary material for: Superconducting gap structure of FeSe
Source: Sci Rep. 2017 Mar 7;7:44024. doi: 10.1038/srep44024 (PMC5339780; doi:10.1038/srep44024)
Supplement: Supplementary Information [file srep44024-s1.pdf]

Supplementary information for
Superconducting gap structure of FeSe

Lin Jiao[1],* Chien-Lung Huang[1],* Sahana Rößler[1],† Cevriye Koz[1],
Ulrich K. Rößler[2], Ulrich Schwarz[1], and Steffen Wirth[1‡]
[1] *Max Planck Institute for Chemical Physics of Solids, Nöthnitzer Straße 40, 01187 Dresden, Germany and*
[2] *IFW Dresden, Postfach 270016, 01171 Dresden, Germany*

(Dated: January 10, 2017)

Abstract

In the supplementary material we present different combinations of s-wave and extended s-wave models used for fitting the specific heat $C(T)$. We used the parameters obtained from the $C(T)$ analysis to simulate the tunnelling spectra using Dynes function. These simulated curves are compared with the symmetrized experimental spectra to exclude some of the models for the gap structure.

*These authors contributed equally
†Electronic address: roessler@cpfs.mpg.de
‡Electronic address: wirth@cpfs.mpg.de

The U-shape (i.e. a finite energy range within which the tunneling conductance goes to zero) of the tunnelling spectra of FeSe presented in the main text gives strong evidence for nodeless superconductivity. Here we show that the specific heat data also can not be described by taking accidental nodes into account. The fittings to these models can be seen in Figs. S1 and S2. In these fittings, the accidental nodes were allowed by constraining the value of anisotropy parameter $\alpha \geq 1$. While in Fig. S1 a single extended s-wave model is considered, in Fig. S2 a sum of s-wave and an extended s-wave ($s+es$) with accidental nodes on the smaller gap is considered. When $\alpha = 1$, the low-temperature shoulder in the fitted curve shifts to the higher values of temperature and deviate strongly from the measured $C(T)$ curve. These forced fits do not represent the experimental $C(T)$ data. Thus, our tunnelling spectroscopy and specific heat measurements support nodeless superconductivity in FeSe.

In the following Figs. S3-S5, we present fitting results of specific heat $C(T)$ with an extended s-wave, two s-wave gaps, and s-wave + extended s-wave models to the Bardeen-Cooper-Schrieffer (BCS) equation [1]. As can be seen in Fig. S3, a single extended s-wave model (without nodes) does not fit to the experimental data too well. However, in addition to the model presented in the main text, the following models, (i) considering two s-waves called model ($s + s$) and (ii) model ($s + es$) with an s-wave and an extended s-wave with the larger gap assigned to the isotropic gap, fit the $C(T)$ data equally well, see Figs. S4, S5.

In Fig. S6, an STM topography containing a twin boundary (TB, the bright stripe in the topography) is presented. The unidirectional behaviour of the electronic dimers can be unveiled from the fact that the orientation of the dimers is independent of the orientation of the impurities but rotates by 90° across the twin boundary.

To confirm that the U-shape of the tunneling spectra as $V_b \to 0$ is a common feature in our samples, we performed STM/STS on a second crystal. In this case we conducted our measurements on an area of 100 $\times$100 nm^2 without any TB, see Fig. S7. As can be seen in Fig. S7(b), even the small gap could be resolved in some cases in the tunneling spectra. Note that there can be some influence of the tunneling tip on the resolution of the spectra, O.P. Sprau et al. [2]. However, within a small range of V_b, the spectra retain a U-shape indicating the absence of low energy quasiparticle excitations owing to finite superconducting gap over the Fermi surface.

In order to identify the correct model, we used the parameters obtained from the $C(T)$

analysis to simulate the tunnelling spectra using a Dynes gap function [3] and compared it with the symmetrized experimental data. Since the simulated curves strongly deviated from the experimentally observed tunnelling spectra (see, Figs. S8-S10), we refitted the STM-data instead with an independent determination of the parameters for the extended s-wave model. However, the fitted curve deviated from the experimental data close to $V_b \to 0$, suggesting a likely presence of a small superconducting gap. In Fig. S11, we compare the results of a single extended s-wave fit as well as a fit to $(s+es)$ model. The latter model fits to the data close to $V_b \to 0$ better. But the coherence peaks of the small gap are rounded in the measured tunnelling spectra. This is likely because the magnitude of the small gap is close to the resolution limits of our STM. However, when the thermal broadening as well as the broadening cased due to the specific type of spectroscopic measurement were included in the fit, the tunnelling data could be reasonably represented by a $(s+es)$ model. In Table SI, all parameters obtained from fitting different models and their root-mean-square deviations $\chi_{\rm rms}$ are presented for comparison.

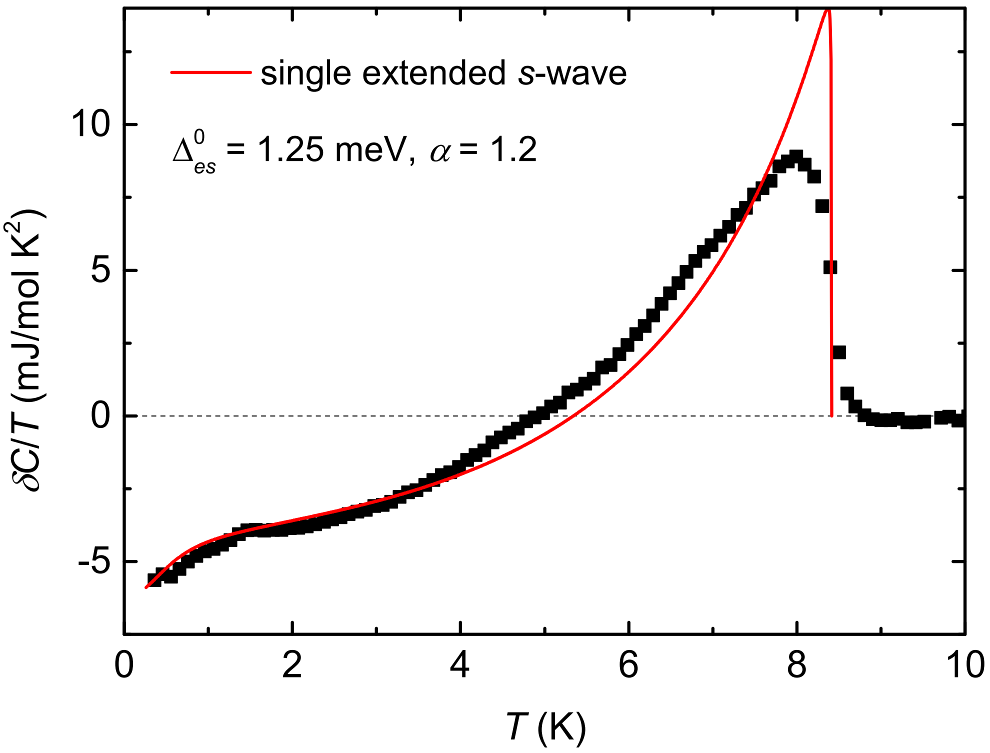

FIG. S1: Zero-field electronic specific heat (with the normal-state specific heat being subtracted) divided by temperature. The solid line represents a fit by an extended s-wave model with parameters $\Delta^0_{es}(0) = 1.25$ meV and $\alpha = 1.2$, where the value of α was constrained to $\alpha > 1$.

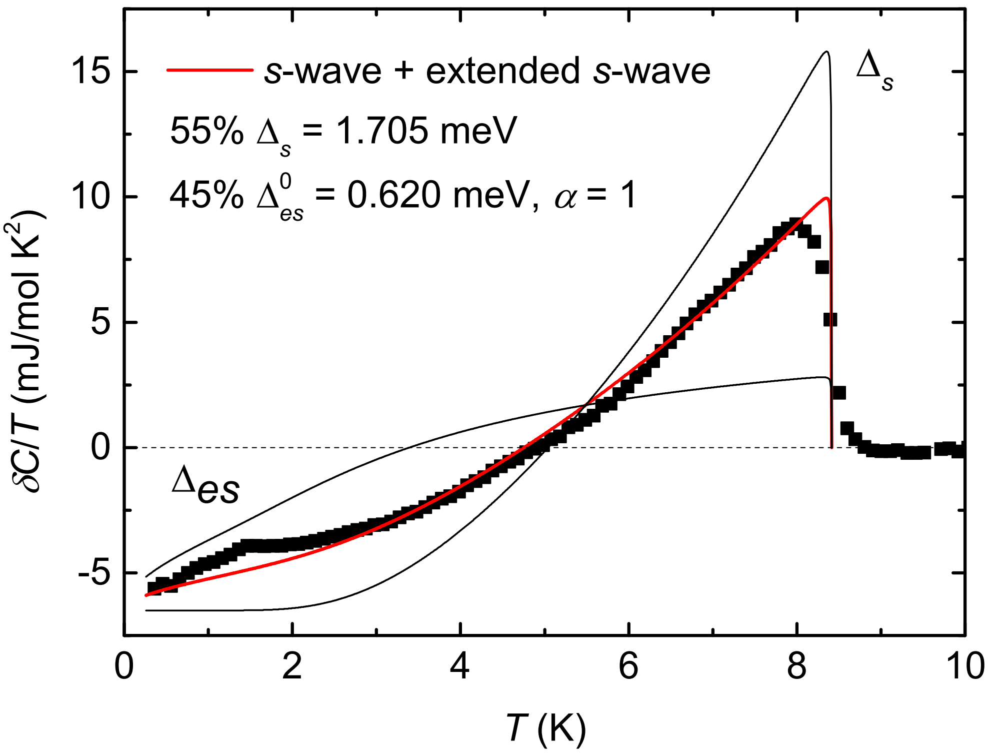

FIG. S2: Zero-field electronic specific heat (with the normal-state specific heat being subtracted) divided by temperature. The solid line represents a fit by taking a sum of a s-wave and an extended s-wave model. Here, $\alpha = 1$ was given as a constraint. The values obtained from the fits for $\Delta^0_{es}(0)$ $= 0.620$ meV and $\Delta_s(0) = 1.705$ meV.

[1] Bardeen, J. Cooper, L. N. & Schrieffer J. R. Theory of superconductivity. *Phys. Rev.* **108**, 1175 (1957).

[2] Sprau, P. O. *et al*. Discovery of orbital-selective Cooper pairing in FeSe. arXiv:1611.02134 (2016).

[3] Dynes, R. C. Narayanamurti, V. & Garno, J. P. Direct measurement of quasiparticle-lifetime broadening in a strong-coupled superconductor. *Phys. Rev. Lett.* **41**, 1509 (1978).

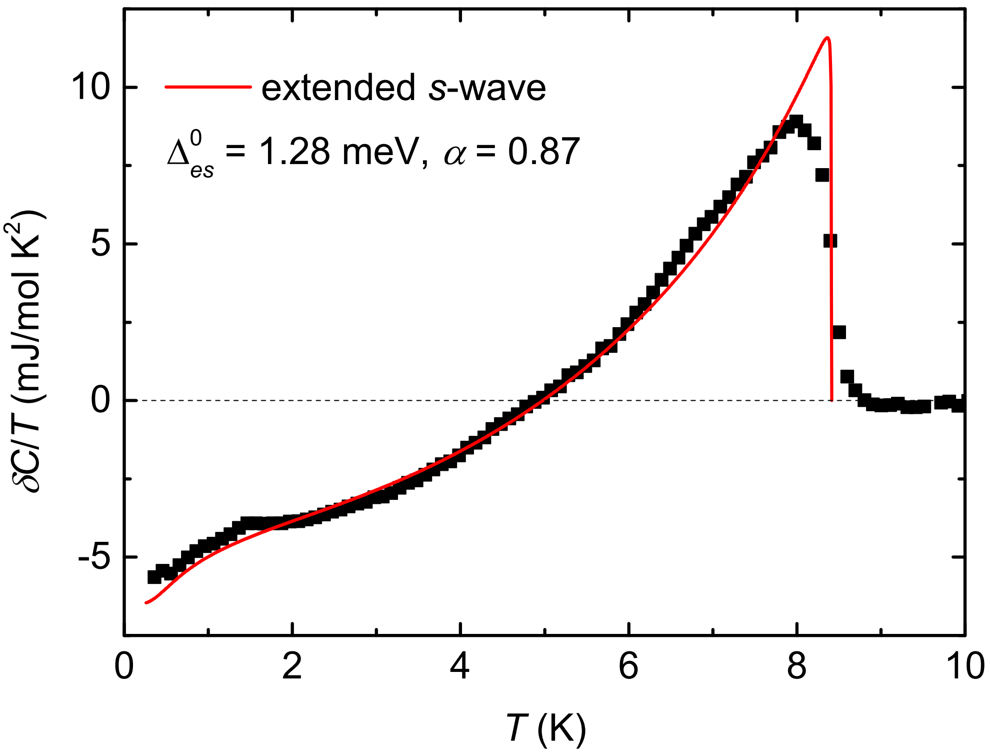

FIG. S3: Zero-field electronic specific heat (with the normal-state specific heat being subtracted) divided by temperature. The solid line represents a fit by an extended s-wave model with parameters $\Delta_{es}^{0}(0) = 1.28$ meV and $\alpha = 0.87$.

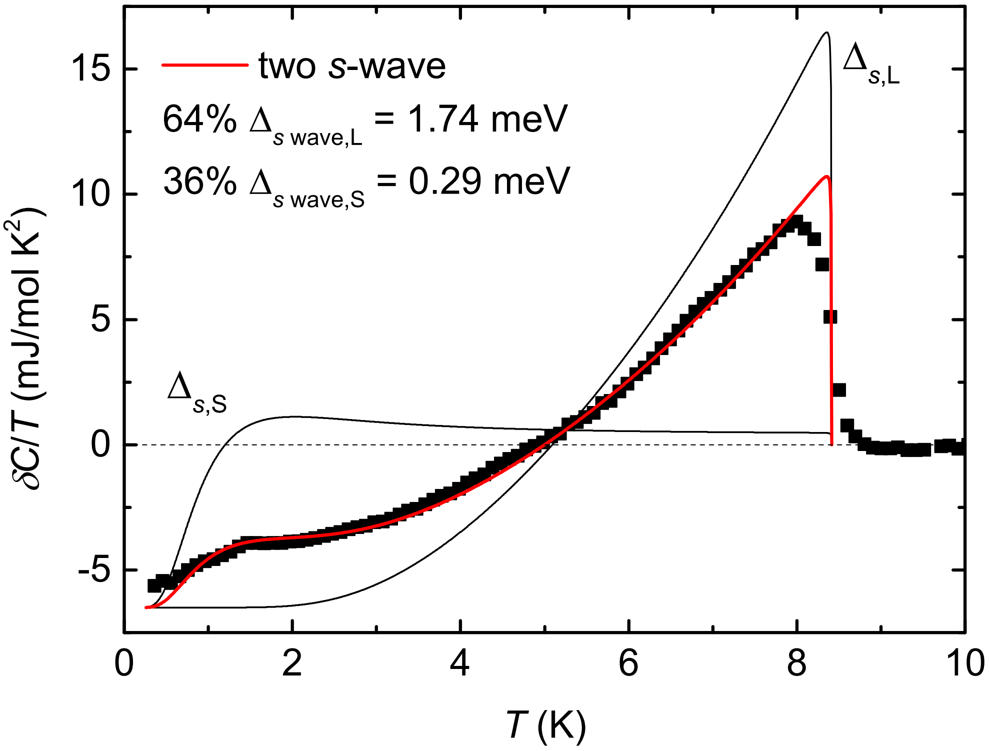

FIG. S4: Zero-field electronic specific heat (with the normal-state specific heat being subtracted) divided by temperature. The solid line represents a fit using a sum of two s-wave-gap models. The large s-wave gap $\Delta_{s,\,\mathrm{L}}(0) = 1.74$ meV and $\Delta_{s,\,\mathrm{S}}(0) = 0.29$ meV.

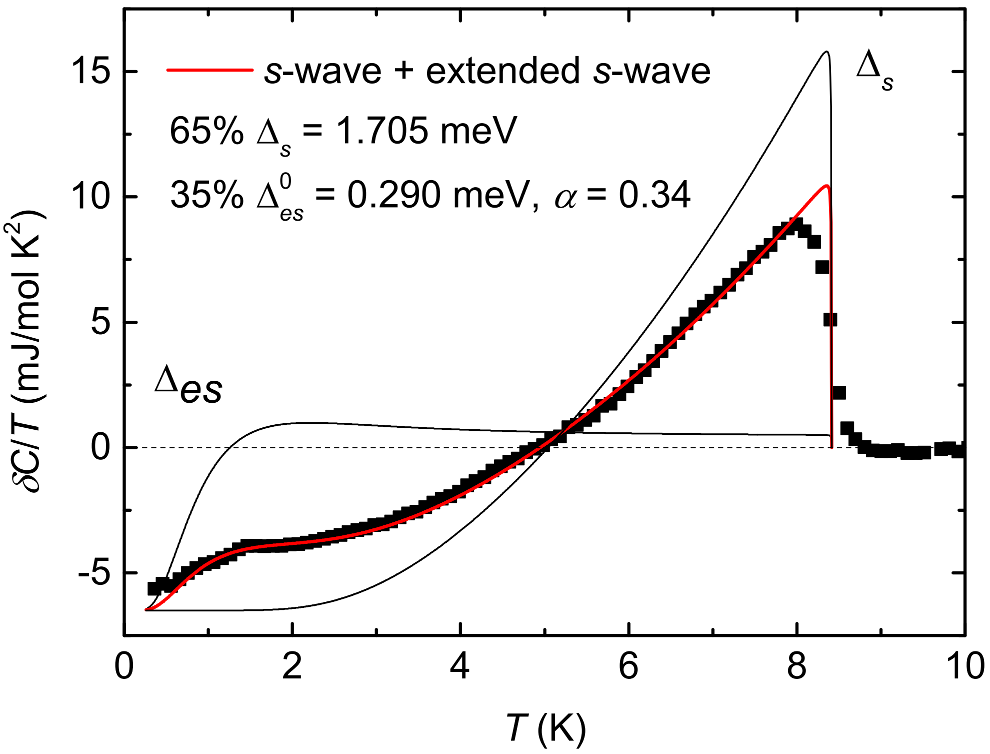

FIG. S5: Zero-field electronic specific heat (with the normal-state specific heat being subtracted) divided by temperature. The solid line represents a fit by a larger s-wave plus a smaller extended s-wave model. The gap values for $T \to 0$ were found to be, for the s wave $\Delta_s(0) = 1.705$ meV, and for the extended s-wave, $\Delta^0_{es}(0) = 0.290$ meV and $\alpha = 0.34$.

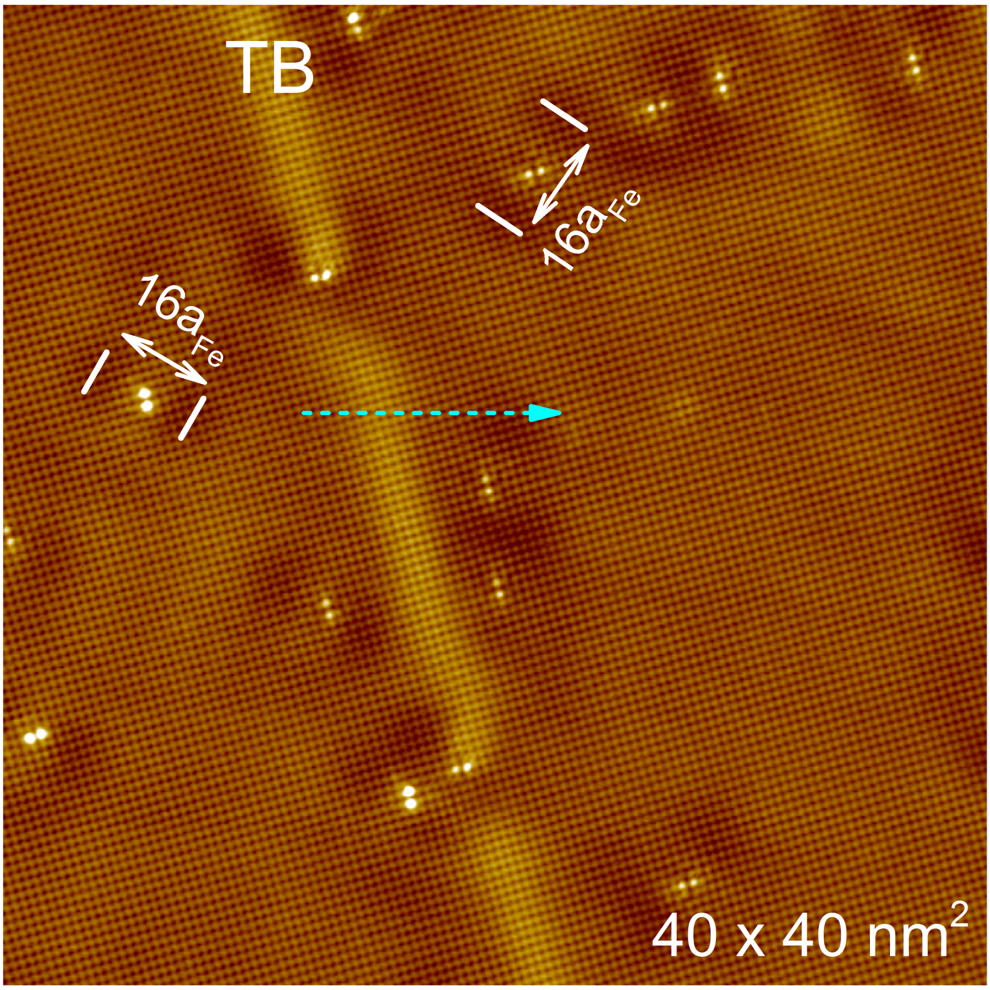

FIG. S6: A topography of FeSe on an area of 40 $\times$ 40 nm^2 obtained at 0.35 K. A twin boundary (TB) appears as a bright region across the topography. The white lines mark the unidirectional electronic dimers of length $\sim$ 16 $a_{\rm Fe}$, where $a_{\rm Fe}$ is the distance of the Fe-Fe atoms in the crystal structure. The cyan arrow marks the region for a line scan measurement of Fig. 5 (main text). The bias voltage and the tunnelling currents were set at V_b = 10 mV and I_{sp} = 100 pA, respectively.

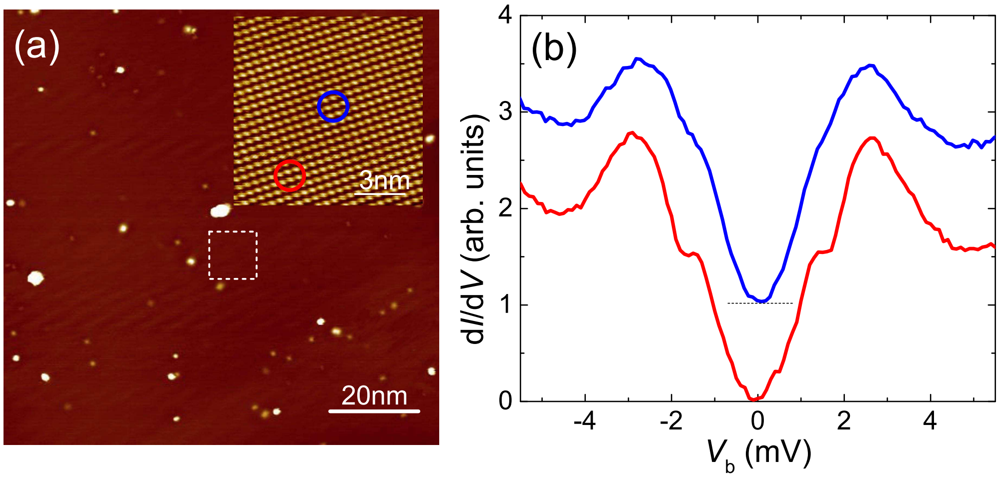

FIG. S7: (a) A topography of FeSe on an area of 100 × 100 nm^2 obtained at 0.35 K. The inset displays a zoomed area marked by the white dashed square. The bias voltage and the tunnelling currents were set at V_b = 10 mV and I_{sp} = 100 pA, respectively. (b) Tunneling spectra taken on two different locations marked in the inset of (a) by red and blue circles.

Table SI: Summary of fitting parameters using different order parameters. Δ is a superconducting gap; W denotes relative weight of gaps; α denotes the gap anisotropy of extended-s-wave; $\chi_{\rm rms}$ is a root-mean-square deviation.

order parameter	Δ_1 (meV)	Δ_2 (meV)	W_1 (%)	W_2 (%)	α	$\chi_{\rm rms,STM}$ [a]	$\chi_{{\rm rms},C/T}$ [b]
single *es*	1.28	—	100	—	0.87	0.731	0.271
single *es*	1.25	—	100	—	1.2	—	0.363
two s-wave	1.74	0.29	64	36	—	0.623	0.278
large s + small *es*	1.705	0.29	65	35	0.34	0.580	0.222
large s + small *es*	1.705	0.62	55	45	1.0	—	0.433
large *es* + small s [c]	1.67	0.25	68	32	0.34	—	0.180
large *es* + small s [d]	1.35	0.6	87	13	0.3	0.062	—

[a]calculated between $V_b = \pm 1.8$ meV
[b]calculated below T = 5 K
[c]best fit for specific heat
[d]best fit for STM

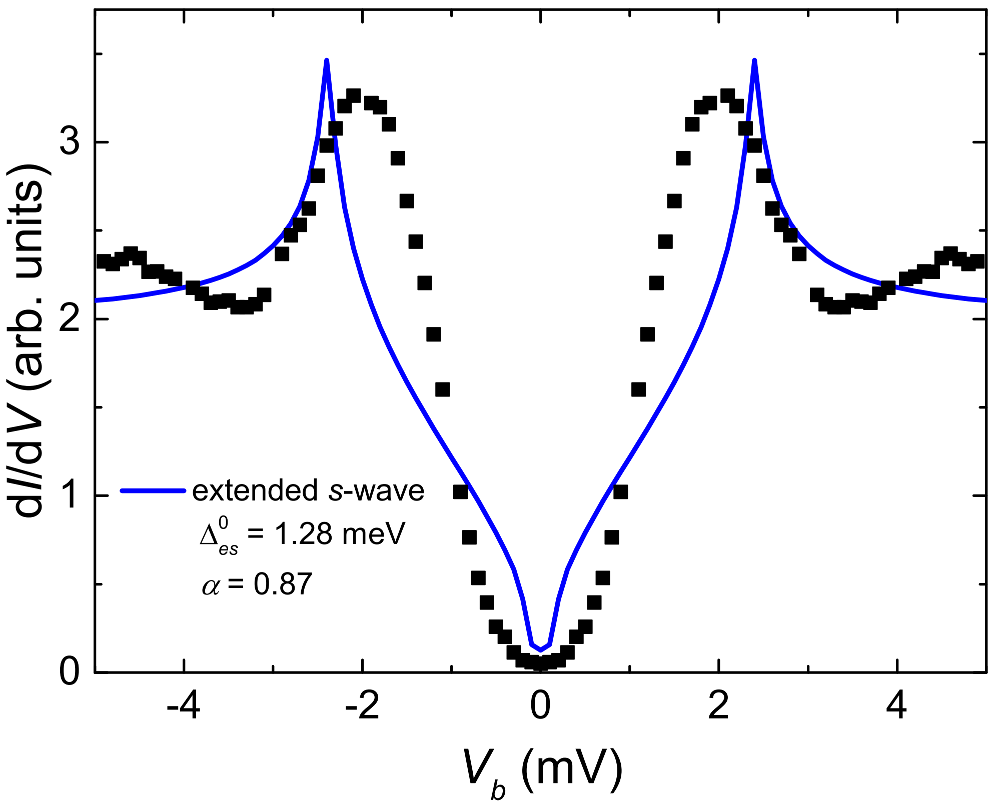

FIG. S 8: A simulated tunnelling spectrum (solid line) using a Dynes gap function. An extended *s*-wave model was used. The simulated curve is compared with the symmetrized tunnelling spectroscopy data taken at 0.35 K.

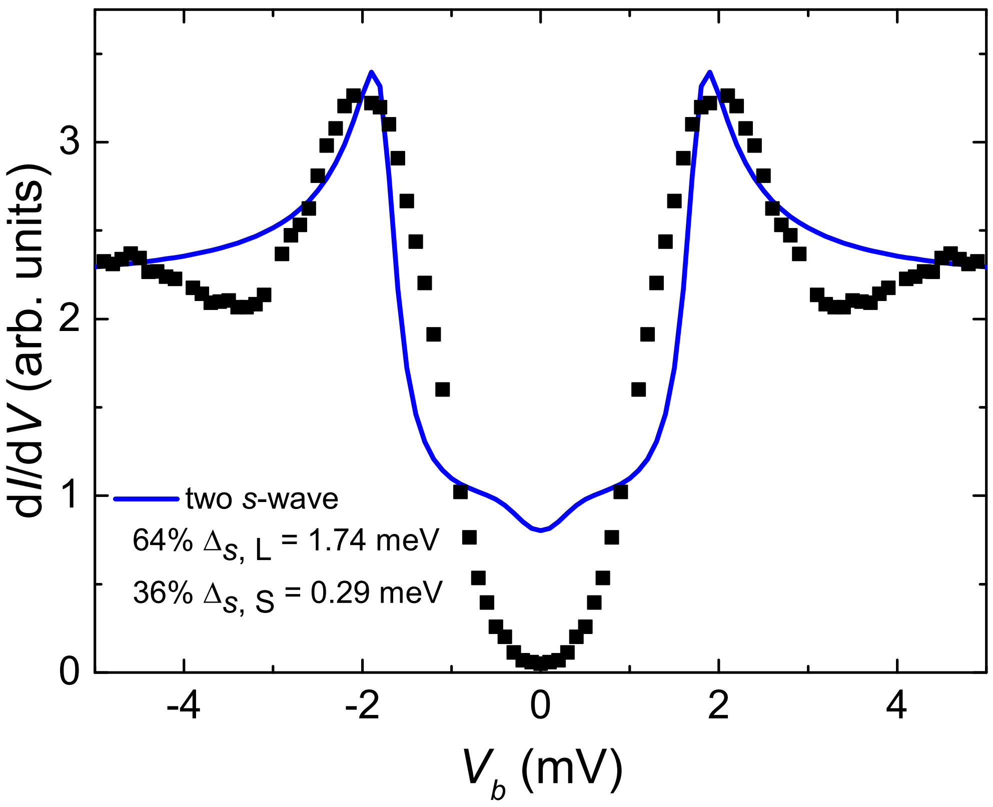

FIG. S 9: A simulated tunnelling spectrum (solid line) using a sum of two Dynes gap function with two different s-wave gaps. The simulated curve is compared with the symmetrized tunnelling spectroscopy data taken at 0.35 K.

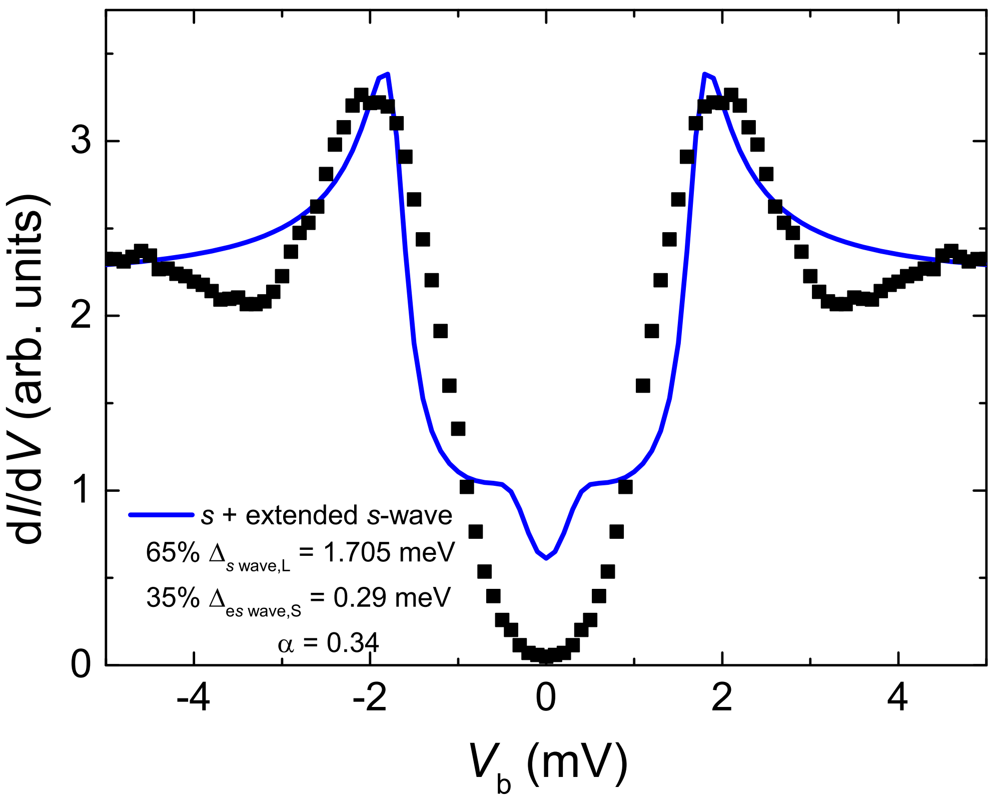

FIG. S10: A simulated tunnelling spectrum (solid line) using a sum of two Dynes gap functions with an s-wave + an extended s-wave models.The simulated curve is compared with the symmetrized tunnelling spectroscopy data taken at 0.35 K.

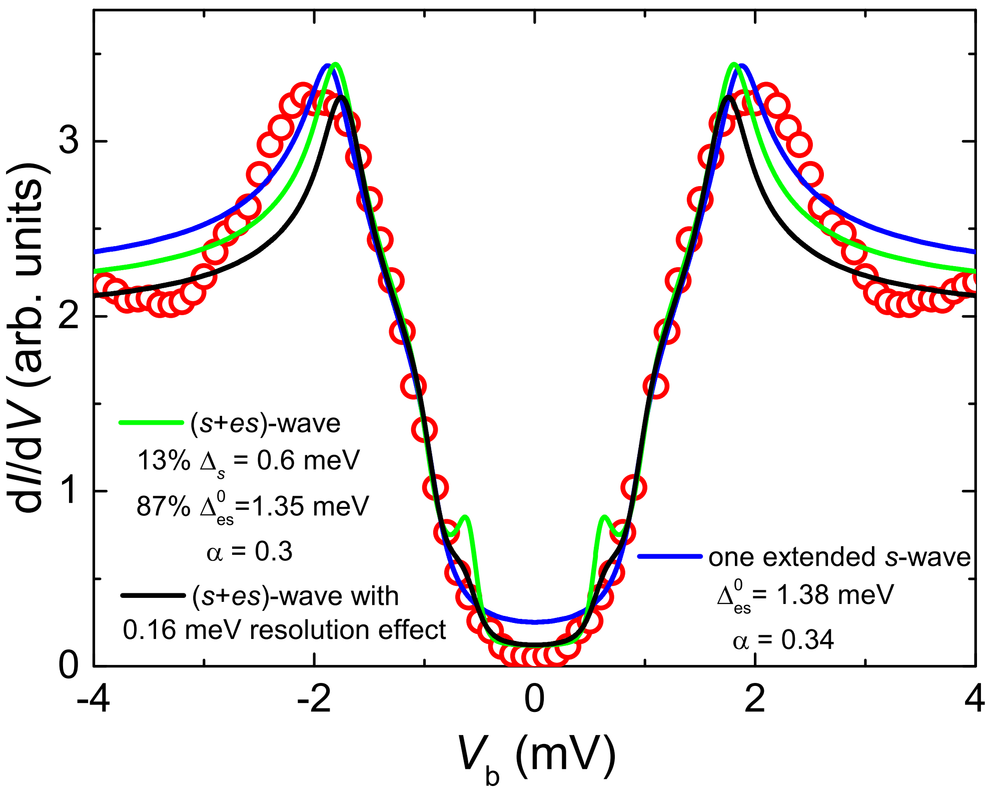

FIG. S 11: A comparison of fittings using a single extended s-wave (Fig. 3 (c), main text) and a sum of two gap functions with an s-wave + an extended s-wave models. The black curve includes the effect of broadening caused by a limited energy resolution of 0.16 meV in the fittings with an s-wave + an extended s-wave model. Note that the gap value obtained from the Dynes function is about the full-width half-maxima (FWHM) of the gap in the dI/dV-spectra, while dI/d$V \approx 0$ holds only within an even smaller energy range around $V_b = 0$
